# De novo pure erythroid leukemia with rapid progression harboring *EZH2* and *TP53* frameshift mutations: A case Report

**DOI:** 10.1097/MD.0000000000039766

**Published:** 2025-05-09

**Authors:** Huanhuan Qin, Xiangyu Li, Zhiguang Lin, Kun Chen

**Affiliations:** a Department of Laboratory Medicine, Huashan Hospital, Fudan University, Shanghai, China; b Department of Hematology, Huashan Hospital, Fudan University, Shanghai, China.

**Keywords:** case report, E-cadherin, NGS, PEL, *TP53* mutation

## Abstract

**Rationale:**

Pure erythroid leukemia (PEL) is a rare and highly aggressive subtype of acute myeloid leukemia. Herein, a rare case of de novo PEL with rapid progression harboring *EZH2* and *TP53* frameshift mutations was reported.

**Patient concerns:**

A 57-year-old male presented with a 3-week history of pancytopenia and peripheral blasts (9%). He also had a 6-day history of hematemesis.

**Diagnoses:**

The case was diagnosed as PEL. Bone marrow examination, immunophenotype, gene mutation analysis, and karyotyping confirmed the diagnosis of PEL.

**Interventions:**

Early in his illness, the patient received acid suppression therapy, gastric protection, hemostatic treatment, and transfusions of red blood cell suspension and platelet concentrates at a local hospital. He was later transferred to our hospital, where the diagnosis of PEL was made, and induction chemotherapy was initiated.

**Outcomes:**

Following chemotherapy, the patient developed granulocytopenia, severe anemia, and thrombocytopenia. He required multiple transfusions of apheresis platelets and red blood cell suspensions for symptomatic relief. However, due to financial concerns, the patient discontinued treatment and passed away 20 days after starting therapy.

**Lessons:**

Due to the unclear pathogenesis of PEL and the lack of targeted therapeutic drugs, the prognosis is inferior. Further research into the signaling pathways regulated by the identified mutations and their potential as therapeutic targets is essential to improve the prognosis of this aggressive form of leukemia.

## 1. Introduction

Pure erythroid leukemia (PEL) is a rare and aggressive subtype of acute myeloid leukemia (AML) and accounts for approximately 1% of all AML diagnoses.^[1]^ However, the 2022 International Consensus Classification (ICC) includes PEL under a broader category of “acute myeloid leukemia with mutated *TP53*.”^[2]^ PEL frequently presents with complex karyotype and *TP53* mutations while its etiology and molecular mechanisms remain unknown. The prognosis for PEL is very poor with a median survival time of only 1.4 to 3 months.^[3]^

PEL can arise very rarely de novo, while more frequently occurring as a therapy-related neoplasm or transformation from myelodysplastic syndromes (MDS).^[4]^ This report presents a unique case of de novo PEL harboring *EZH2* and *TP53* frameshift mutations and rapid disease progression.

## 2. Case report

**Figure 1.**
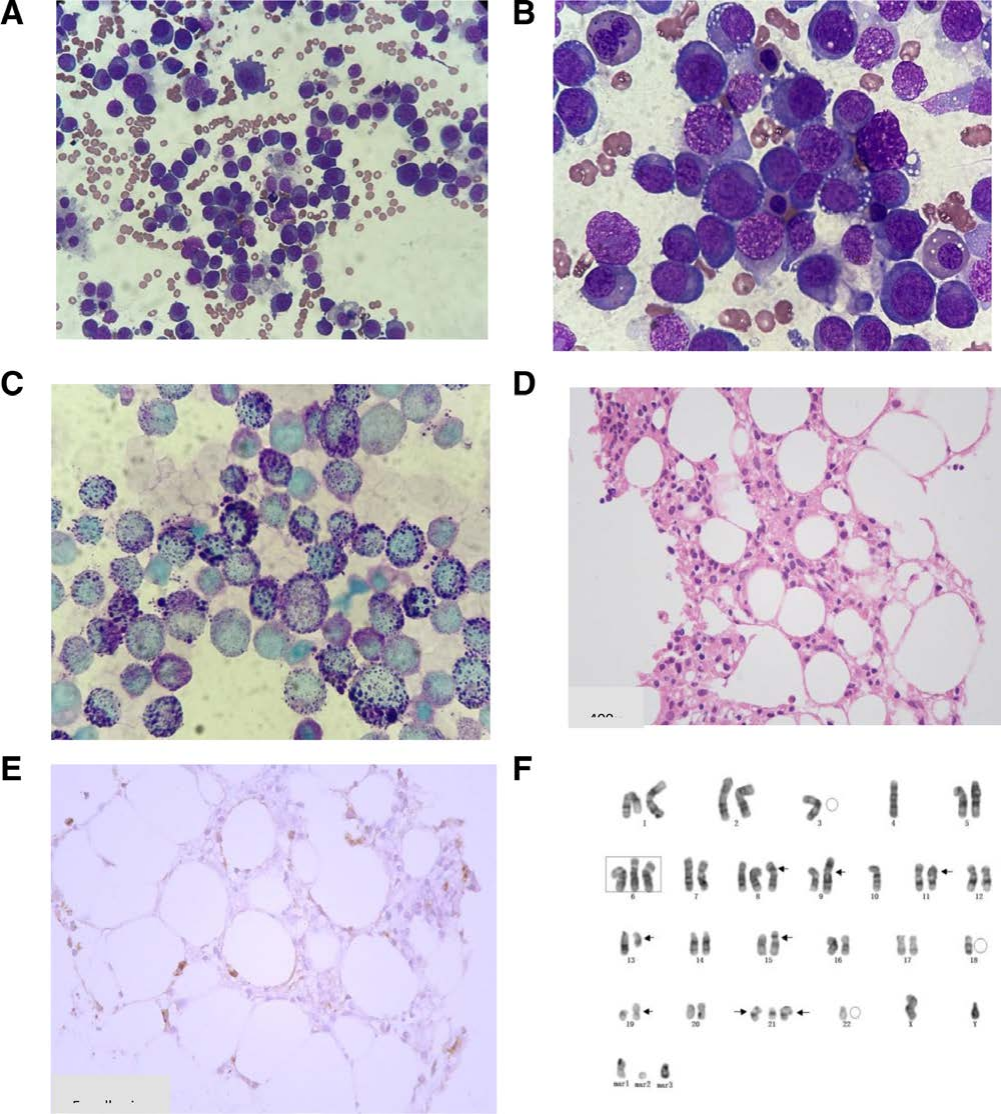
(A) Wright 10× bone marrow smear; (B) Wright 100× bone marrow smear. (C): PAS 100× bone marrow smear. (D) Bone marrow biopsy showing a sheet of blasts with fine chromatin and prominent nucleoli (Wright, 400×). (E) Positive for E-cadherin at 400×. (F) Cytogenetic revealing a complex karyotype.

**Figure 2.**
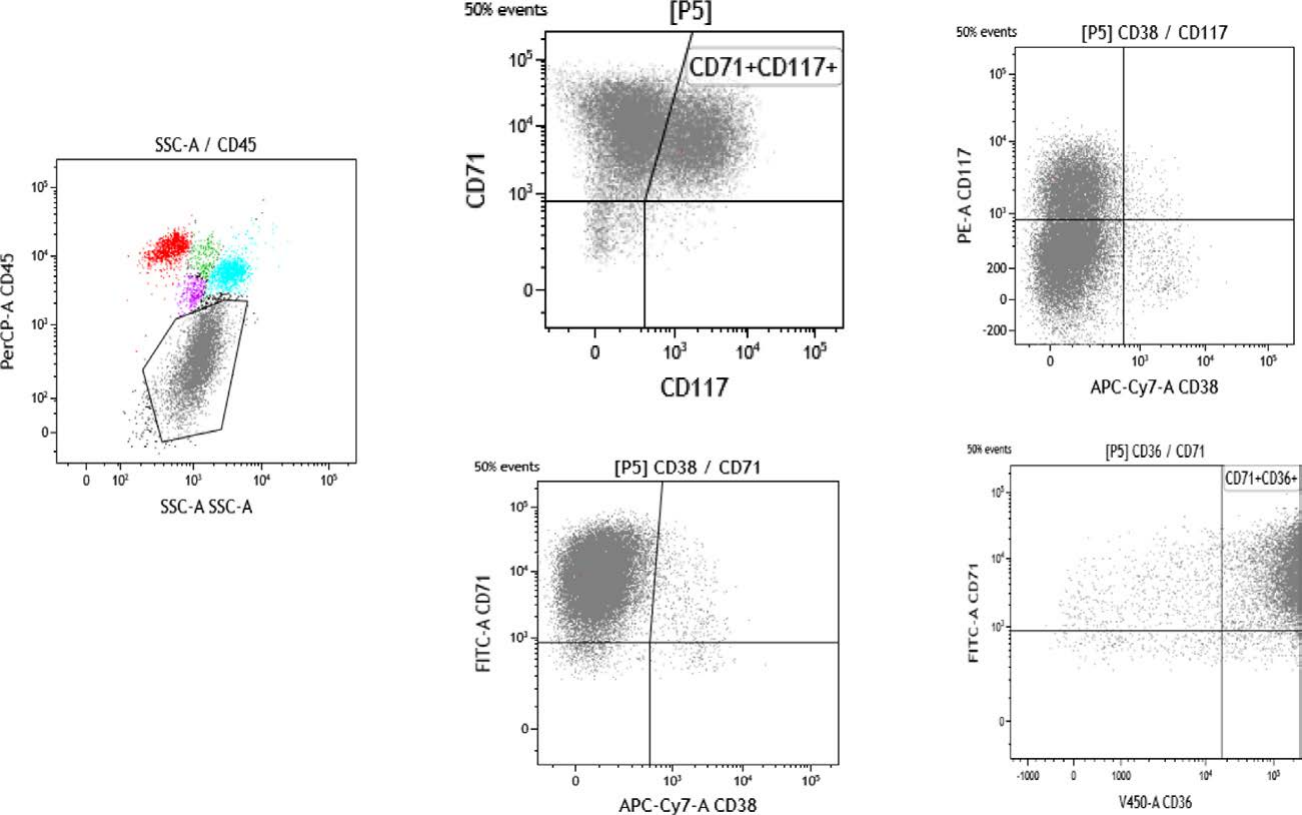
The results of bone marrow flow cytometry: CD45+, CD117+ partly, CD71+, CD38−, and CD36+.

A 57-year-old male patient with acute course, was admitted because of pancytopenia for 3 weeks and 9% peripheral blasts. Laboratory tests revealed anemia (Hb 82 g/L), EPO (1.88 mIU/mL), markedly elevated serum lactate dehydrogenase (LDH1385 U/L), ferritin (1567 ng/mL), D-D dimer (6.58 mg/L), and FDP (10.7 μg/mL) (Table 1). Folic acid and vitamin B1 were normal. He had a 6-day history of hematemesis, with upper gastrointestinal endoscopy revealing superficial gastritis. PET-CT scan showed mild hypermetabolic bone marrow in multiple bones and bilateral multifocal rib uptake. There was also increased FDG uptake in multiple small lymph nodes in the cervical, bilateral hilar, mediastinal, retroperitoneal, and bilateral inguinal regions (SUVmax 3.5). The patient denied night sweats, exposure to toxic chemicals, or radiation. Bone marrow examination revealed 62% of proerythroblasts with high nuclear/cytoplasmic ratio, fine chromatin, distinct nucleoli, basophilic cytoplasm, POX (−) and PAS (+) (Fig. 1C). The blasts exhibited CD45 (+), CD117 (+) partly, CD71 (+), CD36 (+) and CD38 (−) by flow cytometry (Fig. 2) and E-cadherin (+) by IHC staining (Fig. 1E). The PEL diagnosis was made based on histological features (Figs. 1 and 2). Next-generation sequencing analysis revealed negative chimeric screening, *EZH2* (p. Arg81GlyfsTer5fs11.9%), and *TP53* frame shift heterozygous mutations (p. Ser106AlafsTer17fs24.8%) (Table 2). G-banding karyotyping revealed a complex karyotype(38~48,XY,-3,add(4)(q12),add(4)(q12),+6,+8,add(8)(p21),t(9;13)(p13;q12),del(10)(q25),del(11)(p13),der(14)t(10;14)(q11.2;p13),der(15)t(15;21)(p11.2;q11.2),-18,-19,add(19)(p12),+21,add(21)(p11.2),add(21)(p11.2),-22,+mar1[cp13]/46,XY[7]) (Fig. 1F). The bone marrow examination, immunophenotype, gene mutation, and karyotype were consistent with PEL diagnosis. The patient died of rapid disease progression despite induction chemotherapy.

**Table 1:** Patient’s laboratory findings.

Item	Results	Range
WBC	1.92 × 10^9^/L	(3.5–9.5) × 10^9^/L
RBC	2.66 × 10^12^/L	(4.3–5.8) × 10^12^/L
Hb	82 g/L	(130–175) g/L
PLT	12 × 10^9^/L	(125–350) × 10^9^/L
EPO	1.88	5.4 to 31.0 mIU/mL
D-D dimer	6.58	≤0.55 mg/L
FDP	10.7	<0.5 μg/mL
LDH	1385	120 to 250 U/L
PCT	0.38	≤0.05 ng/mL
Fe	4.8	10.6 to 36.7 μmol/L
TIBC	40	40.8 to 76.6 μmol/L
Ferritin	1567	30 to 400 ng/mL
TRF	1.6	2.0 to 3.6 g/L
HBA1c% (NGSP)	6.7%	4% to 6%
IL-2R	737	223 to 710 U/L
IL-6R	13.1	<3.4 pg/mL
CA125	105.7	<35 U/mL

**Table 2:** The results of whole-exome sequencing (myeloid hematology-targeted 248 mutations panels).

Gene	Reference sequence accession number and sequence changes	The frequency of variant (VAF)
*EZH2*	NM_004456.5:c.241del,(p.Arg81GlyfsTer5)	11.9%
*TP53*	NM_00546.5:c.315del(p.Ser106AlafsTer17)	24.8%
*JAK2*	NM_004972.3:c.227-7T > C	48%
*ASXL3*	NM_030632.3:c.1333C > T(p.Pro445Ser)	46.8%
*FANCC*	NM_000136.3:c.35A > G(p.Tyr12Cys)	48.2%
*KSR2*	NM_173598.6:c.1784C > T(p.Pro595Leu)	48.4%
*MECOM*	NM_004991.4:c.1343C > A(p.Thr448Asn)	44.9%
*MGA*	NM_001164273.1:c.5969C > T(p.Thr1990Met)	49%
*RELN*	NM_005045.4:c.9938A > G(p.Gln3312Arg)	48.2%

## 3. Discussion

In this case, a diagnosis of de novo PEL was confirmed through comprehensive investigations, including bone marrow examination, flow cytometry, IHC staining, and genetic analysis. Two frameshift mutations were detected in *EZH2* and *TP53* gene which indicates poor prognosis in myeloid tumors. Notably, the high frequency of *TP53* mutation could imply its role in PEL pathogenesis, potentially contributing to the characteristic genetic instability and complex karyotypes observed in this disease.^[5,6]^ Further supporting the diagnosis, PAS staining revealed strongly positive, coarse cytoplasmic granules (Fig. 1C). The neoplastic cells partially expressed CD33 and CD117 but lacked CD34 and HLA-DR, consistent with previously reported PEL marker profiles.^[7]^ Importantly, other myeloid, T-cell, and B-cell markers were also absent. Flow cytometry detected a significant proportion of nucleated red blood cells (65.43%) in the bone marrow, with 27.3% expressing CD117. Notably, chemotherapy treatment induced an even higher presence of red blood cells in the bone marrow (97.5%), of which 87% were primitive. PEL, unfortunately, is characterized by a clinically aggressive course and currently lacks a standardized treatment protocol, leading to a very poor prognosis for patients.^[5]^ De novo PEL typically presents with pancytopenia, significant bone marrow hypercellularity, a complex karyotype, an aggressive clinical course, and a striking male predominance.^[8]^ While our case did not exhibit chromosomal abnormalities involving chromosomes 5 and 7, frequently observed in PEL,^[9]^ further investigation may be warranted to rule out their presence in other metaphases.

Pure erythroid leukemia (PEL), also known as acute erythroid leukemia (AEL), accounts for approximately 1% of all cases of AML.^[1,10]^ The low prevalence of PEL has unfortunately limited extensive research into its etiology. However, recent advancements have identified frequent *TP53* mutations and p53 overexpression as hallmarks of the disease,^[8,11]^ implicating the *TP53* pathway in its pathogenesis. Unlike other forms of AML, *TP53* alterations are nearly ubiquitous in AEL, reported in almost all PEL cases. Despite this high prevalence, their functional role in disease development remains incompletely understood. While the recent studies by Maria-Riera et al suggest that the *TP53*R248Q mutation primarily confers self-renewal and survival capabilities by repressing known p53 targets, rather than directly impacting erythroid differentiation,^[12]^ complete abrogation of p53 function, often associated with frameshift, nonsense, or splice site mutations, appears to be crucial for full-blown PEL development.^[12]^ In our specific case, the identified *TP53* frameshift mutation leading to null p53 expression potentially represents the underlying molecular mechanism and driving force of PEL development. However, it is important to acknowledge that *TP53* mutations alone might not be sufficient to completely block erythroid differentiation and incite the pathognomonic pronormoblast proliferation characterizing PEL. Other genetic and pathway alterations involved in erythroid differentiation likely play a cooperative role in the disease’s development.^[13]^ For instance, *EZH2* mutations have been linked to aberrant erythropoiesis in patients with primary myelofibrosis (PMF), manifesting as impaired erythroid progenitor maturation and cell cycle arrest.^[14]^ In our case, the comorbidity of *EZH2* mutations with the *TP53* frameshift mutation could have contributed to a more aggressive PEL phenotype with compromised erythroid differentiation.

EPO is a well-characterized cytokine that primarily targets erythroid progenitors and early precursors, stimulating their differentiation and proliferation. In PEL, leukemic cells lose the ability to differentiate and are arrested at the pronormoblast stage. This impairs iron utilization for hemoglobin synthesis, potentially leading to iron-mediated reactive oxygen species formation. In our case, both EPO and iron were found to be significantly deficient. Further investigation into the role of iron accumulation in PEL pathogenesis is warranted, particularly the potential contribution of iron-induced reactive oxygen species to the complex cytogenetic aberrations observed in PEL patients.^[6]^ Previous studies have highlighted the critical role of cytogenetic abnormalities in predicting response to chemotherapy and overall survival in PEL patients.^[9]^ For example, patients with AEL exhibiting an aberrant karyotype demonstrated significantly lower complete remission rates compared to those with a normal karyotype (37% vs 83%).^[15]^ Taken together, these findings suggest that p53 and *E2H2* mutations, EPO and iron deficiency, and complex karyotypes may collectively contribute to the poor prognosis and rapid disease progression observed in PEL.

Due to the rarity of PEL, no standard therapeutic approach has been established. This limitation primarily stems from the scarcity of large-scale prospective trials and the lack of well-defined treatment targets. However, advancements in molecular genetics offer promising avenues for further elucidating PEL pathogenesis, paving the way for more effective treatment strategies for this challenging disease.

## Acknowledgments

All authors contributed to the study’s conception and design. Huanhuan Qin collected the clinical and histological data and drafted the manuscript. We also thank Zhiguang Lin and Xiangyu Li for their assistance with collecting patient’s clinical data. Kun Chen reviewed and approved the manuscript. All authors have read and approved the final manuscript.

## Author contributions

**Conceptualization:** Huanhuan Qin, Xiangyu Li.

**Data curation:** Kun Chen, Huanhuan Qin, Xiangyu Li.

**Formal analysis:** Huanhuan Qin, Xiangyu Li.

**Investigation:** Huanhuan Qin, Xiangyu Li.

**Methodology:** Huanhuan Qin, Zhiguang Lin.

**Resources:** Huanhuan Qin, Zhiguang Lin.

**Software:** Kun Chen, Huanhuan Qin, Zhiguang Lin.

**Supervision:** Kun Chen, Huanhuan Qin, Zhiguang Lin.

**Validation:** Huanhuan Qin.

**Visualization:** Huanhuan Qin.

**Writing – original draft:** Huanhuan Qin.
